# Ways that nursing home nursing staff build resilience: a phenomenographic approach

**DOI:** 10.1186/s12877-022-03582-7

**Published:** 2022-11-16

**Authors:** Sung Ok Chang, Eun Young Kim

**Affiliations:** 1 College of Nursing, and BK21 FOUR R&E Center for Learning Health Systems, Korea University, Seoul, Republic of Korea; 2 Department of Nursing, Soonchunhyang University, Dongnamgu, Soonchunhyang 6Gil 31, 31151 Cheonan, Republic of Korea

**Keywords:** Nursing home, Nursing staff, Resilience, psychological, Qualitative research

## Abstract

**Background:**

Resilience has been studied as an effective concept in nursing that acts as a protective factor which aids in overcoming difficult situations and related mental problems. With the recent increase in demand for nursing homes, nursing home nursing staff are facing a variety of stresses and psychological burdens. Improving resilience has been suggested as one way to deal with the difficulties, such as stress, exhaustion, and burnout, that nursing home nursing staff are experiencing. In order to provide successful education aimed at improving such resilience, it is very important to understand how to perceive experience from the learner’s point of view.

**Aim:**

The study’s aim is to identify the ways that nursing home nursing staff build resilience.

**Method:**

This study used phenomenography, a methodology for exploring the relationship between subject and phenomenon. From January 15, 2022 to February 20, 2022, data collection was undertaken at three nursing homes located in the Republic of Korea. The data was collected through semi-structured interviews with 20 nursing staff members in NHs and a data analysis that strictly followed the 7-step analysis process of phenomenography.

**Results:**

Eight categories were derived. The eight categories were then divided into two groups of four representing perception and strategy. Perception included four categories: ‘grasping the situation’, ‘thinking about one’s responsibility for the resident and personal values’, ‘considering one’s strength’ and ‘thinking of an improved self’. Strategy included four categories: ‘evaluation of oneself and one’s environment’, ‘taking care of oneself’, ‘finding concrete ways to manage the problem’ and ‘self-development for growth’. Perception had three levels of awareness, valuing and assuring, while strategy had three levels of identifying, introspecting and concretizing.

**Conclusion:**

This study provides insight into how individual nursing staff build resilience, a complex and subjective concept. It provides a foundation for future resilience education of nursing home nursing staff and suggests future educational intervention development directions.

**Supplementary Information:**

The online version contains supplementary material available at 10.1186/s12877-022-03582-7.

## Introduction

The steady increase in the global population of older people [1] has been accompanied by a growing interest in nursing homes (NHs), residential facilities for many older people [2]. NH refers to a facility that provides 24-hour functional support and care to people who need assistance with their daily activities and identified health needs, while NH staff refers to those who directly look after residents [3]. Although the composition of the workforce that provides care in NHs varies from country to country, the occupations that account for the largest proportion of nursing staff in Korea NHs are nurses, nursing assistants and care workers [4–6]. With the expanding number of and demand for NHs, the need for nursing staff is also rising, yet the supply of such workers is so far proving insufficient [7]. In addition, existing NH nursing staff are exposed to various difficulties, such as high labor intensity and poor working environments [8]. Accordingly, they are experiencing high levels of stress, job dissatisfaction, and burnout, as well as mental health issues [9, 10]. These problems can lead to missed care, lower nurse well-being, and the perception of lower quality of care [9, 10]. Therefore, it is necessary to think about strategies that can help NH nursing staff overcome the difficulties they face and increase their overall well-being.

The recent COVID-19 pandemic situation has further exacerbated the situation in NHs, with nursing staff experiencing burnout from increased workloads, shortages, severe isolation and the emotional burden of caring for residents facing illness and death [11]. In this context, approaches to support the well-being of health care workers, including nursing staff, have been attempted by improving resilience through education [12, 13].

Resilience has been widely used as a protective factor that helps the subject overcome difficult situations [14, 15] and has been raised as an effective concept that can be important in managing workplace stressors and mental health problems for nurses [16, 17]. It has been studied in various fields, such as psychology, psychiatry, education and social work, and has been defined in various ways. Resilience is sometimes defined as a personal characteristic that helps positive adaptation in difficult situations [18, 19] and has been described as a dynamic process that can be possessed only in the presence of adversity [20]. In a recent study, the concept of resilience of NH nursing staff was found to have the characteristics of coping with situations using internal resources and external support [21]. The meaning of resilience is expanding, and attention is being paid to the importance of building resilience in education, sociology, and public health policy [20, 22, 23]. According to Masten and Obradovic [24], many attributes are involved in the building of resilience, which, it has been argued, is not a single characteristic or process but a complex family of concepts. In addition, several studies have asserted that resilience can be learned as a process of struggling against hardship [25–27].

The importance of education in improving resilience has been continuously emphasized [28]. To date, the effectiveness of a training program for building capacity and resilience for care workers for people with dementia has been shown [29], and various education programs to improve resilience have been developed [27, 28]. However, since education programs reflecting the characteristics of NH nursing staff are not being actively developed, basic research is needed to develop effective education programs.

Various education-related studies of resilience have been conducted, such as educator strategies to strengthen the subject’s internal resources [30, 31], effective teaching methods [32, 33], educators’ roles [34], and curriculum development-related studies for improving resilience [35]. An examination of these studies reveals that an educator-centered perspective on what to teach and how to teach plays a central role. Many viewpoints on how to improve educational effectiveness have been expressed, highlighting the importance of comprehending and assessing the related learning phenomena. Martton [36] suggested focusing on the learner as the subject of experience for understanding more about such learning phenomena. Because each learner has a different way of learning, thinking, and experiencing, it is vital to conceptualize and analyze such phenomena from the learner’s perspective in order to provide successful learning. Consequently, it has been proposed that understanding how learners perceive and experience phenomena can lead to far more effective learning [36, 37].

Ungar emphasized that resilience requires a contextual approach that fits the culture to which the concept belongs [38]. This suggests that the need to explore the nature of how the subject experiences resilience varies depending on the context. Moreover, since resilience includes subjectivity and individual differences can be very large depending on the subject, a suitable method considering these factors is required for an in-depth investigation of resilience.

Phenomenography is a research method that seeks to understand an individual’s experience of a phenomenon and focuses on differences between individuals in their ways of experiencing the world [36, 37]. While phenomenography shares many similarities with phenomenology, the key in phenomenology is to find the essence or most invariant meaning of a phenomenon [39]. On the other hand, in phenomenography, the aim is to find variations in the world experienced rather than only a single essence [40]. By using phenomenography, this study aimed to explore how NH nursing staff build and sustain their resilience. This study can provide foundational data and specific directions for developing future educational programs and various strategies to improve and maintain NH nursing staff’s resilience.

### Aim

The purpose of this study is to identify the ways that NH nursing staff build resilience using a phenomenographic method.

## Methods

### Design

This study used phenomenography to identify the ways of being resilient of NH nursing staff. Phenomenography is a methodology that studies the relationship between subject and phenomenon, exploring how the subject experiences the world with a focus on differences in perception based on individual experiences [36, 41, 42]. Therefore, it was considered appropriate for the purpose of this study and was thus selected. This study complied with the Consolidated Criteria for Reporting Qualitative Studies (COREQ) guidelines [43].

### Researchers’ experience and training

The first author of this study is a professor at a university nursing college and is an expert in qualitative research. She has a wealth of experience in conducting phenomenography-related research, providing her with a sufficient understanding of phenomenographic research. The corresponding author of this study is a university research professor who completed qualitative research classes in her doctoral program and has conducted a number of studies using various qualitative research methods. The corresponding author is interested in the resilience of NH nursing staff and is continuously conducting related research. At the stage of starting this study, she thoroughly reviewed the previous research that had applied the phenomenographic method to become knowledgeable in this research method. Researchers kept a self-reflection diary during the study period and regularly recorded their experiences, reactions, assumptions, biases, and perceptions during the study period [44, 45]. Through self-reflection, the researchers made continuous efforts to eliminate bias and maintain neutrality.

### Data collection

Data collection was conducted from Jan 15, 2022 to Feb 20, 2022. It was carried out at three NHs located in Seoul, Korea. The three NHs accommodate 300–400 residents with dementia and/or comorbidities. Two are government-run facilities and the other is run by a private nurse. We used purposive sampling because the key criterion in selecting research participants in phenomenography is whether the participants possess enough experience to represent the phenomenon of interest rather than the sample’s size [37, 41]. The researchers posted a recruitment notice for nurses, nursing assistants and care workers containing the purpose and explanation of the study on the bulletin boards of the three NHs for 1 month. Among the subjects who voluntarily expressed their intention to participate, those who met the inclusion criteria for this study were selected and the researchers contacted them individually. Subjects who were not selected as study participants were individually contacted and the reasons for their not having been selected were explained. The researchers first identified the attributes of resilience through a literature review. The attributes identified through the literature were positivity, patience, mindfulness, supportive relationships, resources available, work-life balance, self-growth and self-management. After the managers of three domestic NHs were explained the attributes of resilience revealed through the literature review and the phenomenon to be identified through this study, they selected suitable subjects from among the care workers, nursing assistants, and nurses at their facilities.

For in-depth conversations in phenomenography, it is recommended to present specific scenarios related to the research concept and phenomenon to be explored prior to an interview in order to help the interviewee understand the research concept [46]. Giving participants time to read the scenario before their interviews can help participants understand the phenomenon [46]. The three scenarios used in this study were created through discussion by two of the study’s researchers with the purpose of describing three levels (high, medium and low) of the resilience of NH nursing staff (Table 1. Scenarios demonstrating the resilience of nursing home nursing staff). Two experts, a professor of gerontological nursing and a NH manager, verified whether the contents of the scenarios were appropriate. Participants were not informed in advance of the individual scenario’s resilience levels (high, moderate, or low), and how they perceived the difference was confirmed through interviews. The specific interview guidelines are presented in Supplementary File 1. As the interviews progressed, participants were free to move to topics of interest, interview questions were flexibly changed according to the content of the conversation, and not all participants were asked all the questions in the guidelines. Data were collected through semi-structured interviews, which are a data collection method suitable for phenomenography [47].

**Table 1:** Scenarios demonstrating the resilience of nursing home nursing staff

***Instructions****: Please read the following three scenarios and rate them for high, medium, and low resilience. After you have read all the scenarios, the researcher will ask you questions about them.*	
1 (High)	Ms. Park (female/32 years old), who is a care worker and has worked for 6 years at a nursing home, has recently had the number of patient care tasks she has to perform increased due to the resignations of her co-workers. Because she has to work 2 hours overtime every day, she is having a very hard day physically. She thought about quitting her job several times a day, but she couldn’t because of her concern for the patients she would leave behind. She felt comforted when she talked to like-minded co-workers about the difficult situation. She also talked to her manager about a problem she didn’t think she could solve and asked for the manager’s help. Through these actions, Ms. Park’s desire to quit her job subsided, and she decided to work harder, thinking of the people who helped her.
2 (Medium)	Mr. Kim (male/35 years old) is a nursing assistant who has worked at a nursing home for 5 years. The facility’s manager recently changed, and Mr. Kim was always being scolded by the new manager. Mr. Kim was afraid of being scolded in front of his co-workers, and he wanted to quit his job. He became unsure about caring for patients, and he felt that he was unqualified to be a nursing assistant. However, his colleagues comforted him and sympathized with him. He appreciates his co-workers’ efforts, but he thinks he’ll quit 1 day because he’s become obsessed with the idea that he’s not the right person for nursing home work.
3 (Low)	Ms. Lee (Female/28 years old) is a nurse who has been working in a nursing home for 8 months. Ms. Lee did her best to take care of the residents, but recently discovered that one of the patients she took care of had pressure sores on the tailbone. A week ago, she received from the patient’s daughter a severe complaint about the patient’s pressure sores. Mr. Lee felt guilty about the patient’s worsening condition contrary to her intentions, and she felt depressed as she began to think she was unqualified to be a nurse. Eventually, she resigned and left the nursing home.

Since phenomenography does not set an appropriate number of study participants, the number of participants in previous studies varied from 5 to 90 [48, 49]. In phenomenography, the number of participants is determined by the saturation of the data rather than the number of people [50]. Data saturation is achieved when no new data turns up in the data collection [51].

The interviews were conducted face-to-face. The interview setting was the patient education room in the NH. The interviews were recorded with the consent of the participants. The interviews were conducted individually and were recorded using the recording function of the researcher’s smartphone. When no new data were revealed, it was judged that data saturation was reached and the interviews were stopped. After the interview, the interview contents were transcribed, and detailed information such as the atmosphere of the site and the tone of voice, facial expression, and mood of the participants was recorded in field notes using a laptop computer. Twenty participants were interviewed once and additional interviews were conducted when the researcher determined that additional information collection was necessary during the analysis process. Finally, 9 participants were interviewed once and 11 participants were interviewed twice. The average interview time was 55.8 minutes. The interview transcripts were returned to the participants so they could check the contents for accuracy. Additional interviews were then conducted for clarity of meaning, for a total of 42 interviews, all of which were conducted by the corresponding author.

### Data analysis

The theoretical foundation of phenomenography is a phenomenological interpretive approach [36]. Therefore, the validity of phenomenography is determined not by how accurately the result reflects the objective reality, but how persuasively the result describes the phenomenon [52]. To ensure such a study’s validity, the seven-step analysis process of phenomenography [53] is strictly followed as a procedure, and the logic of the descriptive category and output space derived from the analysis process is confirmed [52]. Accordingly, to logically derive and describe the descriptive categories and output spaces of the nature of experience of the resilience of NH nursing staff, Marton’s [36] phenomenography research method was used, and each step was faithfully performed. The data analysis process is presented in detail in ‘Table 2. The process of data analysis’.

**Table 2:** The process of data analysis

The 7 steps of data analysis in phenomenography	
1. Familiarization	This is the stage in which the empirical data that has been collected by the researcher is considered. The researchers transcribed the audio recordings of 20 participants and read them repeatedly until they could clearly perceive the statements.
2. Condensation	This step involves an intensive reading of the transcribed material to identify its most important elements. The researchers found commonalities and differences by classifying and organizing meaningful sentences or important statements using color highlighters.
3. Comparison	Potential concepts are identified and then compared to each other to discover their intrinsic characteristics. The researchers then proceeded with coding by deriving tentative concepts centered on simplified semantic sentences and keywords. In this process, researchers tried to derive concepts by focusing on context rather than direct language. Two researchers independently extracted codes and then compared their results; 36 codes were extracted through the process of reaching consensus.
4. Grouping	Organize groups that represent preliminary classifications of similar answers.
5. Articulating	Establish clear boundaries between categories. The researchers carried out the process of confirming the exact meanings and correcting the categories through additional interviews to resolve any ambiguous or unclear content.
6. Labeling	Give the category an appropriate name.
7. Contrasting	Check the concordance between the finally derived categories.

During this process, we reached agreement through discussions with two qualitative research experts. Each analysis step was analyzed independently by the same researchers as above, and consensus was reached through discussion at each step. The analysis results were confirmed by the participants to increase the validity of the analysis, and debriefings were performed between researchers was performed during the analysis process to increase the reliability of the study.

### Ethical consideration

The investigation was approved prior to the start of the study and followed the principles specified in the Declaration of Helsinki. This study was reviewed and approved by the Korea University Institutional Review Board (KUIRB-2021-0384-01). The researchers fully explained the purpose and method of the study to each participant before the start of the study and obtained informed consent. It has been explained that personal information will be protected, that the information obtained will be used only for research and that it will be destroyed after a certain period. All participants voluntarily consented to participate in the study.

## Findings

The participants consisted of 10 nurses, 6 nursing assistants, and 4 care workers. Their ages ranged from in the 30s to 60s, and their average work experience in an NH was 8.12 years. All the participants were women (Table 3. General characteristics of the participants (*N* = 20)).

**Table 3:** General characteristics of the participants (*N* = 20)

Characteristics	N (%)	Mean (SD)	
Occupation			
Nurse	10 (50.0)		
Nursing assistant	6 (30.0)		
Care worker	4 (20.0)		
Age (years)			
30–39	3 (15.0)	52.75 (10.05)	
40–49	4 (20.0)		
50–59	10 (50.0)		
60–69	3 (15.0)		
Work experiences in NH (mean, years)			
1–5	8 (40.0)	8.12 (9.15)	
6–10	9 (45.0)		
10–15	3 (15.00)		
Gender			
Women	20 (100)		
Men	0		
Education			
College	15 (75.0)		
Master’s degree	5 (25.0)		

As a result of analyzing the collected data, the 36 codes derived from the analysis process were grouped into 8 categories, which were then divided into two groups: perception, which represents a cognitive process and strategy, which represents a process for transitioning to concrete implementation. Perception confirmed the process in which the subjects in difficulty became aware of the situation, thought about their responsibility to the resident and their own values, and became confident about problem-solving while considering their inner strength and growth. These categories were divided into three levels—awareness, valuing and assuring—after the researcher’s deliberation process. Strategy consisted of the process of evaluating oneself and one’s environment to discover the possibility of problem-solving, taking care of oneself first so as to remain strong, and finding a way to solve problems and grow one step further. The four categories belonging to strategy were also divided into three levels—identifying, introspecting and concretizing—through the researcher’s analysis process.

### Perception of the ways NH nursing staff demonstrate resilience

Four categories were found under the perception of resilience of NH nursing staff: grasping the situation, thinking about one’s responsibility for the resident and personal values, considering one’s strengths, and thinking of an improved self.

#### Category P1: grasping the situation

When NH nursing staff were faced with a difficult situation, they first tried to find out what the situation was and its cause(s). They sought to determine whether the difficulty was caused by some specific situation, the environment, or themselves. Having awareness of the situation was crucial for setting their future direction in problem-solving.

“There is too much work and too much complexity going on around me. In any difficult situation, the most important thing is to know why it happened. If I don’t recognize the reason, it will mix with other situations, and I won’t be able to identify it [Participant 3, nurse].”

#### Category P2: thinking about one’s responsibility for the resident and personal values

When the NH nursing staff found themselves in a difficult situation, they thought of the residents and worried about the residents who would not be cared for. Since their job is to provide care for residents, they tried to do their best to fulfill their duties, even when they were difficult. Additionally, they recalled their values and beliefs while determining what they ought to do.

“Due to the resignation of a coworker, I was forced to work excessive overtime. There were many days when I didn’t want to go to work, but all I could think about were the faces of our patients. I pity them if I don’t show up for work. The patients do nothing wrong. I persevere and overcome every time I think of my patients [Participant 5, nurse].”

“Since I was a child, I’ve been trained to be responsible, that whatever I do carries with it the burden of accountability. And, because having fun is crucial in any work, I strive to laugh and have fun when I’m having a bad day. It is quite effective [Participant 12, care worker].”

#### Category P3. Considering one’s strength

Nursing home nursing staff strove to uncover their strengths in tough situations. They believed this was their fundamental ability to overcome obstacles and were sure that they could do so on their own.

“As I faced numerous challenges, I recognized that responding to difficult events one by one would be more difficult. These trying times strengthened me. With patience, I look after my inner self. This approach allows me to see the situation objectively and concentrate entirely on myself [Participant 7, nurse].”

#### Category P4. Thinking of an improved self

Nursing home nursing staff found that developing and improving themselves was very helpful in overcoming difficult situations. They encouraged themselves not to give up, envisioning themselves as better off in the future. They studied more about older people and nursing, tried to show a more developed image, and thought about how to provide better caring to residents. To accomplish that, they thought they had to study and learn more. In addition to these occupational perspectives, they continued to strive to remain physically and mentally healthy human beings.

“Inner growth, in my opinion, is attained via the process of overcoming adversity. Furthermore, I believe that the desire to grow, especially in the face of adversity, is the driving force behind issue solutions. To overcome obstacles, I believe that a goal must be set and that the objective must be forward-looking. I used to make a lot of mistakes when I was caring for patients, and I was angry because I thought I didn’t have any qualifications. I thought at the time that I should learn more about nursing and expand my expertise [Participant 19, nurse].”

### Strategy for supporting the resilience of NH nursing staff

It was identified that the strategy behind resilience of NH nursing staff included four categories: Evaluation of oneself and one’s environment, taking care of oneself, finding concrete ways to manage the problem, and self-development for growth.

#### Category S1. Evaluation of oneself and one’s environment

For problem-solving purposes, the NH nursing staff evaluated themselves and their surroundings. They analyzed themselves and tried to figure out the extent to which they could solve their problems. In addition, they tried to check their level of problem-solving abilities by understanding the environment, such as whether their environment was suitable for solving problems, what help and support they could receive in their environment, and whether their environment was safe.

“It’s important to know what level of problem-solving abilities I have, whether I’m in a supportive setting, and what kind of assistance I can get. I won’t be able to solve anything if it is beyond my capabilities. First and foremost, I must gain a better understanding of myself and my environment in order to establish concrete plans for the future. [Participant 11, nursing assistant]”.

#### Category S2. Taking care of oneself

The NH nursing staff respected themselves and believed that in order to get through the crisis, they first needed to take care of themselves. Through doing so, they attempted to improve their inner strength. They looked back on their prior experiences and sought to develop their strengths with the belief that they could overcome things on their own. Furthermore, they bolstered themselves with positive energy to avoid being overcome.

“There was a moment when the patient’s family was quite upset with me because I was not taking proper care of the patient, and I was hurt. I was crying a lot and having a terrible time, but whenever I’m exhausted, I remember that period. Even when things are difficult, I strive not to cry. I try to keep my mind in check so that I don’t injure myself or that my heart becomes too hard. I believe in taking care of my inner self and strengthening it to prevent it from collapsing [Participant 1, nurse].”

#### Category S3. Finding concrete ways to manage the problem

The NH nursing staff devised specific solutions to the situation. They devised their own approaches for dealing with stress and common problems while developing bonds with their coworkers. Work-life balance was achieved by clearly separating work and personal life. These practical methods directly aided them in resolving their problems.

“I try not to think about work when I get home. At home, I take time to unwind and heal while focusing only on myself [Participant 20, nursing assistant].”

“Conversations with coworkers provide me great relief. To relieve stress, I normally get together with my coworkers after work and talk for a while. After discussing the tense situation, I begin to believe that serious work is not such a big deal. That is why I enjoy socializing with my coworkers [Participant 2, care worker].”

#### Category S4. Self-development for growth

The NH nursing staff devised ways toward personal growth based on the development of a stable life. They didn’t merely consider how to deal with emergencies and challenges, but instead aspired to live a life of gradual development. They attempted to live lives that progressed by gaining information and expertise while also discovering particular strategies for improving themselves. In addition, they had concrete plans for the future.

“For the future, I have particular plans. Life planning, in my opinion, is similar to constructing the skeleton of a building. No matter how difficult things are right now, if I have a clear strategy and vision, I will be on the correct track one day [Participant 17, nurse].”

“I’m studying and learning a lot in the hopes of one day becoming a nursing facility administrator. In two years, I intend to attend graduate school. It’s a lot of fun to devote time to my personal development [Participant 10, nurse].”

### Outcome space

**Fig. 1:**
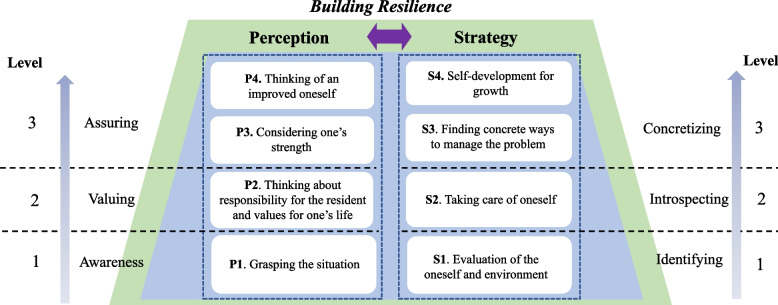
Outcome space expressing the categories of the ways that nursing home nursing staff builds resilience

In phenomenography, findings are described as categories and relationships between categories and are expressed as outcome spaces [54, 55]. An outcome space can be divided into a referential aspect meaning ‘how’ and a structural aspect meaning ‘what’. The referential aspect describes the general meaning of the phenomenon, while the structural aspect describes how each concept relates hierarchically [56]. The outcome space of this study was identified by broadly dividing it into perception and strategy (Fig. 1 Outcome space expressing the categories of the ways that nursing home nursing staff builds resilience). The referential aspects of perception were identified as awareness, valuing and assuring, and the referential aspects of strategy were found to be identifying, introspecting and concretizing. The structural aspect was confirmed from the relationships between the eight categories, with each category representing a hierarchical structure linked with a referential aspect. In regard to the structural aspect in perception, NH nursing staff showed the characteristics of understanding their own situations and thinking about their responsibilities for residents and their life values based on this. These provide support for thinking about their strengths and growth and give them the confidence that they can solve problems and overcome adversity. In the structural aspect of the strategy, the possibility of problem solving is discovered while evaluating oneself and the environment, while by taking care of themselves, they establish a stable foundation on which they can stand. Through this process, they find concrete ways to overcome difficult situations.

## Discussion

This study identified the ways that NH nursing staff build resilience, and it was confirmed that those ways are distinguished by perception and strategy. In perception, they sought to understand the situation, took responsibility for the resident, and thought about the value of life. They thought about their strengths and how they could improve themselves. In their perception of ways of being resilient, they strove to overcome obstacles while thinking about their obligations to residents and the value of their own lives. A previous study that analyzed the experiences of NH nursing staff identified that NH nursing staff felt a high sense of responsibility due to the low functional ability of NH residents and thought a great deal about ways to increase the residents’ independence [57]. In addition, referring to how nurses’ resilience is strengthened by close relationships with residents and sharing experiences [58], it can be seen that nurses’ forming of resilience is positively affected through relationships with residents. In particular, it was reported that they experienced conflicting values in order to provide high-quality care without infringing upon the dignity of residents [59]. These prior studies support the results of this study, and these characteristics appear to be reflected in the resilience of NH nursing staff. In addition, NH nursing staff consider their strengths and think of how to improve themselves. This process can be seen as a process of acquiring the confidence that they can overcome difficult situations and improve their skills and knowledge. The attributes of self-management, leadership, passion for work and positivity presented in previous studies that examined the perception of resilience of nurses caring for older people were confirmed in this study [60]. However, this study went one step further and was able to confirm how the subject built resilience. In strategy, NH nursing staff look internally to contemplate growth in the process of overcoming difficult situations. In the same context, a previous study that explored the resilience experience of nurses found that nurses developed self-esteem by valuing their profession and wanting to develop their professional selves for a more rewarding future [61].

Two other approaches emphasized in strategy were taking care of oneself and actively looking for concrete ways to solve problems. Among the strategies shown in the results of this study, evaluating oneself and one’s environment in strategy, can be interpreted as confirming one’s own capabilities and resources to help them overcome adversity. This basic work of the strategy is also the basis for the next strategy. In the strategy, taking care of oneself, various strategies such as mindfulness, meditation, and finding one’s strength have been suggested in previous studies [62]. Nursing home nursing staff found ways to solve problems on their own and concretized strategies. They demonstrated a self-directed attitude in problem-solving and in developing strategies for growth.

Various studies have been presented to show that education has a positive effect on improving resilience [27, 28]. The present study can be used to inform an education programme that supports the resilience of nursing home nursing staff.

An important characteristic of the perceptions shown in this study is that the NH nursing staff felt a sense of responsibility for their residents, which led them to deal positively with adversity and became a driving force for growth. Another characteristic of the strategies presented in this study is that by taking care of oneself, one strengthens the inner self, and based on this, finds specific methods to manage the problems and grows. These findings provide important evidence for developing programs that improve resilience.

The strength of this study is that, by analyzing phenomena that are difficult to understand and by schematizing the experiences of the subjects, it enables the complexities of the participants’ experiences to be read and comprehended in a practical structure.

There are some limitations in this study. First, the researchers identified the attributes of NH nursing staff resilience through a literature analysis before data collection and attempted to select participants with the identified attributes. Nursing staff who exhibited such attributes were recommended as study participants by NH managers, who were well aware of their employees’ characteristics. However, a limitation is that the NH managers’ subjectivity may have influenced their recommendations and thus the selection of the study’s subjects. In the future, this limitation could be solved by using a tool that measures resilience to help select study participants. Second, since each country implements a different NH system, the occupational composition of an NH nursing staff may differ by country, so it may be difficult to explore and understand the consolidated phenomena by country. In order to overcome these limitations, it is suggested that studies be conducted in various countries in the future.

## Conclusion

Resilience is a complex and subjective concept; hence, studying various perspectives concerning it is essential to enrich resilience-related knowledge. This study, which presents ways of being resilient from the subject’s perspective, can provide practical guidelines for education focused on improving resilience of NH nursing staff.

## Supplementary Information

**Additional file 1.**

## Data Availability

All data generated or analyzed during this study is included in this published article.
