# Supplementary material for: Ways that nursing home nursing staff build resilience: a phenomenographic approach
Source: BMC Geriatr. 2022 Nov 16;22:861. doi: 10.1186/s12877-022-03582-7 (PMC9667428; doi:10.1186/s12877-022-03582-7)
Supplement: Supplementary file 1 — Additional file 1. [file 12877_2022_3582_MOESM1_ESM.docx]

Supplementary file 1. Interview guidelines

|  | ***Examples of interview questions*** | ***Rationale*** |
| --- | --- | --- |
| ***Step 1*** | A reading material of the prepared scenario is provided to the participants.  Wait until the participants have read the reading material sufficiently, and then begin to ask questions.  *“Please summarize what the scenario you read is about.”*  *“Please explain the differences between the scenarios”*  *“What did you discover in the scenario?”*  *“Do you think each scenario includes resilience? What was your reason for thinking that way?* | Reading materials are presented before the interview, giving participants an opportunity to think about learning naturally through reading tasks (Marton & Booth, 1996). |
| ***Step 2*** | Q1. Introduction questions  *“Have you ever faced a difficult situation and overcame it?”*  *“What happened to the episode you mentioned?”* | Start a conversation and move on to a main topic (Kvale, 1996). |
|  | Q2. Follow-up questions & Probing questions  *“Can you please explain in more detail?”*  *“Can you give a more detailed explanation of what happened?”*  *“Can you give a more detailed example of this?*” | To draw a more detailed explanation and a more complete explanation (Kvale, 1996). |
|  | Providing silence | Allow silence to allow the interviewee to think sufficiently to associate and reflect.  Allow the interviewee to break the silence with important information (Kvale, 1996). |
| ***Step 3*** | Q3. Deep exploration of the meaning of sub-components  *“Then you mean that….”*  *“Is it correct to feel that ...?”*  *“Does the expression... cover what you just expressed?”* | Rephrasing the interviewee's responses for clarity and interpretation (Kvale, 1996).. |
| ***Step 4*** | Interview summary and closing  *“Is there anything more you would like to say?”* | Ensure that participants have answered the topic comprehensively and that no further comments are made. |

Marton, F., & Booth, S. (1997). Learning and awareness. Hillsdale, NJ: Lawrence Erlbaum

Kvale,S. (1996), Interviews: an introduction to qualitative research interviewing, Sage, Thousand Oaks, CA.
